# Infectious Disease Physician Availability and Postgraduate Antimicrobial Stewardship Education in Japan

**DOI:** 10.1001/jamanetworkopen.2024.4781

**Published:** 2024-03-29

**Authors:** Toshiki Miwa, Koh Okamoto, Yuji Nishizaki, Yasuharu Tokuda

**Affiliations:** 1 Department of Infectious Diseases, The University of Tokyo Hospital, Tokyo, Japan; 2 Department of Infectious Diseases, Graduate School of Medical and Dental Sciences, Tokyo Medical and Dental University, Tokyo, Japan; 3 Division of Medical Education, Juntendo University School of Medicine, Tokyo, Japan; 4 Muribushi Okinawa Center for Teaching Hospitals, Urasoe, Okinawa, Japan; 5 Tokyo Foundation for Policy Research, Tokyo, Japan

## Abstract

This cross-sectional study evaluates resident physician perceptions of antimicrobial stewardship education in Japan in the presense of infectious disease physicians educators.

## Introduction

Given insufficient knowledge or unpreparedness regarding antimicrobial use among resident physicians,^1,2,3^ current postgraduate antimicrobial stewardship (AS) education may be insufficient. Providing education is an important role of infectious disease (ID) physicians,^4^ but data regarding their contribution to AS education among resident physicians are limited. We aimed to investigate the association between ID physicians’ engagement in postgraduate education and resident physicians’ perceptions and attitudes to elucidate the role of ID physicians as educators.

## Methods

This cross-sectional study used a nationwide Japanese In-Training Examination platform for all resident physicians regardless of their desired specialty in 2023.^5^ All participants who responded to the additional survey were included in this study (eTable in Supplement 1). Univariable and multivariable analyses were performed to assess the associations between resident physicians’ perceptions and attitudes toward antimicrobials and (1) the presence of ID consultation at an institutional level or (2) ID physicians’ education for resident physicians at an individual level. Statistical tests were considered significant if the 2-tailed *P-*value was less than .05 and were performed using Stata version 16 (StataCorp) software (eMethods in Supplement 1). This study was approved by the institutional review board of the Japan Institute for Advancement of Medical Education Program and the University of Tokyo Hospital and followed the Strengthening the Reporting of Observational Studies in Epidemiology (STROBE) reporting guidelines. Data were analyzed in March 2023.

## Results

Among 9011 resident physicians in 633 hospitals who participated in the examination, 4051 (45.0%) from 577 hospitals responded to the survey. Of those, 1256 (31.0%) were female, 2092 (51.6%) were postgraduate year (PGY)–1 physicians, 1959 (48.4%) were PGY-2 physicians, and the median (IQR) age was 26 (26–27). Compared with those working in hospitals without an ID department, respondents working in hospitals with bedside ID consultations were more likely to know the concepts of AS (641 of 1558 [41.1%] vs 532 of 1770 [30.1%]; odds ratio [OR], 1.63; 95% CI, 1.41-1.88; *P* < .001) and more likely to be confident in choosing appropriate antimicrobials (372 of 1566 [23.8%] vs 330 of 1767 [18.7%]; OR, 1.36; 95% CI, 1.15-1.60; *P* < .001) or in choosing narrow-spectrum agents (773 of 1560 [49.6%] vs 750 of 1772 [42.3%]; OR, 1.34; 95% CI, 1.17-1.53; *P* < .001). However, the association was not significant when adjusted for other factors (Table 1). In contrast, the presence of AS programs was consistently independently associated with these items. Regarding ID physicians’ education, multivariable analysis (Table 2) showed that ID department rotation was independently associated with being able to explain the concept of AS (adjusted odds ratio [aOR], 1.68; 95% CI, 1.19-2.37), being confident in choosing appropriate antimicrobials (aOR, 2.21; 95% CI, 1.46-3.36), and trying to choose narrow-spectrum antimicrobials (aOR, 1.94; 95% CI, 1.42-2.66) when compared with receiving no education from ID physicians during residency. Similarly, receiving feedback through ID consultation was independently associated with being able to explain the concept of AS (aOR, 1.49; 95% CI, 1.08-2.06) and with trying to choose narrow-spectrum antimicrobials (aOR, 1.42; 95% CI, 1.06-1.91).

**Table 1.** Adjusted Odds Ratios (OR) of Institutional Factors Associated With Resident Physicians’ Perceptions and Attitudes Regarding Antimicrobial Stewardship (Multilevel Logistic Regression Analysis)^a^

Variable	Participants, No.	OR (95% CI)		
		Presence of AS programs	Type of ID consultation	
			Medical record–review based ID consultation^b^	Bedside ID consultation^b^
Perceptions of AS and antimicrobial resistance				
I agree that antimicrobials potentially do patients harm	3437	1.51 (1.27-1.81)^c^	0.81 (0.61-1.08)	0.82 (0.62-1.09)
I know when to use antimicrobials for asymptomatic bacteriuria	3410	1.42 (1.18-1.71)^c^	0.86 (0.63-1.16)	1.14 (0.86-1.51)
I know when to suspect *Clostridioides difficile* infection	3397	1.79 (1.48-2.17)^c^	0.62 (0.45-0.85)^c^	0.96 (0.71-1.30)
I can explain what AS is	3396	1.88 (1.54-2.31)^c^	0.77 (0.56-1.07)	0.95 (0.70-1.29)
I am confident choosing appropriate antimicrobials	3403	1.28 (1.01-1.63)^c^	0.75 (0.50-1.13)	0.75 (0.51-1.11)
I know when I can choose oral antimicrobials	3385	1.57 (1.29-1.92)^c^	0.77 (0.56-1.06)	0.90 (0.67-1.22)
I know the need for antimicrobial de-escalation	3394	1.94 (1.60-2.36)^c^	0.76 (0.55-1.06)	0.98 (0.72-1.34)
I know appropriate duration of therapy is determined in some infections	3382	2.06 (1.71-2.47)^c^	0.73 (0.54-1.00)^c^	1.00 (0.74-1.33)
Attitudes toward AS and antimicrobial resistance				
I make sure if a patient has allergic history before prescribing antimicrobials	3431	1.65 (1.36-2.02)^c^	0.68 (0.49-0.95)^c^	1.06 (0.77-1.46)
I make sure if a patient is colonized with resistant pathogens before prescribing antimicrobials	3430	1.66 (1.39-1.99)^c^	0.79 (0.59-1.06)	0.87 (0.66-1.15)
I make sure if a patient has antimicrobial exposure before prescribing antimicrobials	3426	1.62 (1.36-1.93)^c^	0.73 (0.55-0.97)^c^	0.91 (0.70-1.19)
I order culture tests when I suspect an infection which needs cultures	3407	2.00 (1.63-2.47)^c^	0.74 (0.52-1.05)	1.18 (0.83-1.67)
I try to choose narrow-spectrum antimicrobials whenever possible	3402	1.80 (1.50-2.16)^c^	0.85 (0.64-1.14)	0.85 (0.65-1.12)
I try to choose oral antimicrobials rather than intravenous ones whenever possible	3380	1.40 (1.17-1.67)^c^	0.86 (0.64-1.15)	1.08 (0.82-1.41)
I try to de-escalate antimicrobials when culture results are back	3385	1.92 (1.60-2.34)^c^	0.77 (0.56-1.06)	1.18 (0.87-1.61)
I try to reduce the duration of therapy as recommended in the guidelines	3382	1,62 (1.36-1.93)^c^	0.77 (0.58-1.02)	1.13 (0.87-1.49)

Abbreviations: AS, antimicrobial stewardship; ID, infectious diseases.

^a^ The model includes sex, postgraduate year, university or community hospital, presence of ID department at medical school, ID department rotation at medical school, AS education at medical school, types of ID consultation during residency, resident physicians’ individual exposure to ID physicians during residency, the presence of opportunity for AS education during residency, and the presence of AS programs.

^b^ No ID consultation was used as reference.

^c^ *P* < .05.

**Table 2.** Adjusted Odds Ratios (OR) of Individual Factors Associated With Resident Physicians’ Perceptions and Attitudes Regarding Antimicrobial Stewardship (Multilevel Logistic Regression Analysis)^a^

Variables	Participants, No.	OR (95% CI)		
		Lectures only^b^	Feedback through ID consultation^b^	ID department rotation^b^
Perceptions of AS and antimicrobial resistance				
I agree that antimicrobials potentially do patients harm	3437	0.85 (0.63-1.15)	1.01 (0.75-1.36)	0.80 (0.58-1.10)
I know when to use antimicrobials for asymptomatic bacteriuria	3410	1.12 (0.82-1.52)	1.03 (0.76-1.40)	1.27 (0.92-1.75)
I know when to suspect *Clostridioides difficile* infection	3397	0.81 (0.58-1.12)	1.31 (0.93-1.85)	0.88 (0.62-1.27)
I can explain what AS is	3396	1.39 (1.00-1.94)^c^	1.49 (1.08-2.06)^c^	1.68 (1.19-2.37)^c^
I am confident choosing appropriate antimicrobials	3403	1.02 (0.66-1.55)	1.44 (0.96-2.16)	2.21 (1.46-3.36)^c^
I know when I can choose oral antimicrobials	3385	1.12 (0.81-1.55)	1.37 (1.00-1.88)	1.48 (1.06-2.06)^c^
I know the need for antimicrobial de-escalation	3394	0.86 (0.61-1.21)	1.06 (0.75-1.50)	0.87 (0.60-1.26)
I know appropriate duration of therapy is determined in some infections	3382	1.05 (0.76-1.44)	1.25 (0.90-1.73)	0.95 (0.67-1.35)
Attitudes toward AS and antimicrobial resistance				
I make sure if a patient has allergic history before prescribing antimicrobials	3431	0.89 (0.62-1.25)	0.79 (0.55-1.12)	0.82 (0.57-1.20)
I make sure if a patient is colonized with resistant pathogens before prescribing antimicrobials	3430	1.08 (0.80-1.46)	1.36 (1.01-1.84)^c^	1.58 (1.15-2.19)^c^
I make sure if a patient has antimicrobial exposure before prescribing antimicrobials	3426	0.79 (0.59-1.06)	1.25 (0.93-1.67)	1.20 (0.88-1.64)
I order culture tests when I suspect an infection which needs cultures	3407	0.78 (0.54-1.13)	0.92 (0.63-1.35)	0.73 (0.48-1.09)
I try to choose narrow-spectrum antimicrobials whenever possible	3402	0.96 (0.71-1.30)	1.42 (1.06-1.91)^c^	1.94 (1.42-2.66)^c^
I try to choose oral antimicrobials rather than intravenous ones whenever possible	3380	0.86 (0.64-1.16)	1.10 (0.82-1.48)	1.16 (0.85-1.59)
I try to de-escalate antimicrobials when culture results are back	3385	0.72 (0.52-1.01)	1.03 (0.74-1.45)	0.81 (0.56-1.16)
I try to reduce the duration of therapy as recommended in the guidelines	3382	0.91 (0.68-1.23)	1.22 (0.91-1.65)	1.08 (0.79-1.49)

Abbreviations: AS, antimicrobial stewardship; ID, infectious diseases.

^a^ The model includes sex, postgraduate year, university or community hospital, presence of ID department at medical school, ID department rotation at medical school, AS education at medical school, types of ID consultation during residency, resident physicians’ individual exposure to ID physicians during residency, the presence of opportunity for AS education during residency, and the presence of AS programs.

^b^ No education was used as reference.

^c^ *P* < 0.05.

## Discussion

In this nationwide cross-sectional study in Japan, at the institutional level, the presence of a bedside ID consultation was associated with resident physicians’ enhanced commitment to AS, although its association was not observed after adjusting for the presence of AS programs. However, at the individual level, ID department rotation or feedback through ID consultation were independently associated with improved perceptions and attitudes toward AS among resident physicians. ID physicians have been reported to exert a positive influence on peer physicians’ antimicrobial prescription behavior, both indirectly at the institutional level, through cultivating the organizational culture, and directly at the individual level.^6^ Our findings further support the value of ID physicians as educators for resident physicians, particularly when education takes place through patient care.

The limitations include the presence of unmeasured factors, such as differences in the quality, timing, and duration of AS education and respondents’ motivation to learn AS. Also, resident physicians’ prescribing practice was not evaluated. Nevertheless, our findings suggest that empowerment of ID physicians in postgraduate education may complement AS programs and help promote AS in the long term.

## Notes

Supplement 1. **eTable. **Survey Questions and Possible Response Categories
eMethods.

eReferences.

Supplement 2.
Data Sharing Statement
