# Supplementary material for: Infectious Disease Physician Availability and Postgraduate Antimicrobial Stewardship Education in Japan
Source: JAMA Netw Open. 2024 Mar 29;7(3):e244781. doi: 10.1001/jamanetworkopen.2024.4781 (PMC10980957; doi:10.1001/jamanetworkopen.2024.4781)
Supplement: Supplement 1. — eTable. Survey Questions and Possible Response Categories eMethods. eReferences. [file jamanetwopen-e244781-s001.pdf]

Supplemental Online Content

Miwa T, Okamoto K, Nishizaki Y, Tokuda Y. Infectious disease physician availability and postgraduate antimicrobial stewardship education in Japan. *JAMA Netw Open*. 2024;7(3):e244781. doi:10.1001/jamanetworkopen.2024.4781

eTable. Survey Questions and Possible Response Categories
eMethods.
eReferences

This supplemental material has been provided by the authors to give readers additional information about their work.

© 2024 Miwa T et al. *JAMA Network Open.*

eTable. Survey Questions and Possible Response Categories

Survey questions	Response categories
**Exposure to ID education during medical school**	
Were there any department providing ID consultation in your medical school?	1. ID department<br>2. Department of infection control<br>3. No<br>4. Unknown
Did you experience ID department rotation?	1. Yes<br>2. No (I did not choose ID department rotation)<br>3. No (None of departments provided ID rotation)
Did you have an opportunity to learn AS?	1. Yes<br>2. No
**Exposure to ID education during residency**	
Does ID department exist in your hospital?	1. Yes (bedside ID consultation)<br>2. Yes (chart-review based ID consultation)<br>3. No
How do you describe your interaction with ID physicians in your hospital?	1. I rotated ID department<br>2. I received feedback through ID consultation<br>3. I took lectures only<br>4. No interaction
Do you have an opportunity to learn AS?	1. Yes<br>2. No
Do AS programs exist in your hospital?	1. Yes<br>2. No<br>3. Unknown
**Perceptions of AS and antimicrobial resistance**	
I agree that antimicrobials potentially do patient harm.	1. Strongly disagree<br>2. Disagree<br>3. Undecided<br>4. Agree<br>5. Strongly agree
I know when to use antimicrobials for	1. Strongly disagree

© 2024 Miwa T et al. *JAMA Network Open.*

asymptomatic bacteriuria.	2. Disagree<br>3. Undecided<br>4. Agree<br>5. Strongly agree
I know when to suspect *Clostridioides difficile* infection.	
I can explain what AS is.	
I am confident choosing appropriate antimicrobials.	
I know when I can choose oral antimicrobials.	
I know the need for antimicrobial de-escalation.	
I know appropriate duration of therapy is determined in some infection.	
**Attitudes toward AS and antimicrobial resistance**	
I make sure if a patient has allergic history before prescribing antimicrobials.	1. Strongly disagree<br>2. Disagree<br>3. Undecided<br>4. Agree<br>5. Strongly agree
I make sure if a patient is colonized with resistant pathogens before prescribing antimicrobials.	
I make sure if a patient has antimicrobial exposure before prescribing antimicrobials.	
I order culture tests when I suspect an infection which needs cultures.	
I try to choose narrow-spectrum antimicrobials whenever possible.	
I try to choose oral antimicrobials rather than intravenous ones whenever possible.	
I try to de-escalate antimicrobials when culture results are back.	
I try to reduce the duration of therapy as recommended in the guidelines.	

Notes

Abbreviations: ID, infectious diseases; AS, antimicrobial stewardship

© 2024 Miwa T et al. *JAMA Network Open.*

eMethods

Study setting and study participants

This cross-sectional study utilized data from the General Medicine In-Training Examination (GM-ITE) platform, which was conducted in January 2023 across Japan. The GM-ITE is a computer-based two-hour examination designed to analyze resident physicians' performance. Its data have been used for medical education-related research purposes [1-5]. The GM-ITE platform is operated by the Japan Institute for Advancement of Medical Education Program (JAMEP), a nonprofit organization that evaluates the quality of medical education in Japan. Each teaching hospital independently determined whether or not to participate in the examination.

The academic year in Japan begins in April and ends in March. Since 2004, Japan has adopted a 'super-rotation' system, where rotation through various departments is mandatory for resident physicians regardless of their desired expertise [6]. There are >18,000 resident physicians in >1,000 teaching hospitals, comprising approximately 120 university hospitals and 900 community hospitals that provide residency across Japan [7,8]. The resident physicians work to complete their residencies at their main hospital, and at other relevant hospitals and clinics. The infectious diseases (ID) department rotation is elective.

Data source

For this study, we developed an original survey concerning perceptions and attitudes regarding antimicrobials based on the knowledge-attitude-practice (KAP) model [9] (eTable 1). A pilot survey was conducted beforehand with resident physicians at the University of Tokyo Hospital to obtain feedback concerning clarity and to ensure the survey's validity. The type of clinical services provided in ID department consultations were categorized as either bedside consultations or chart review-based consultations where ID physicians do not examine patients directly. The type of education that resident physicians received from ID physicians was classified into four mutually exclusive categories: ID department rotation, feedback through consultation services, lectures only, and no education. Items questioning perceptions or attitudes were rated on a five-point Likert scale, ranging from one (strongly disagree) to five (strongly agree). Following GM-ITE completion, we collected demographic data and questionnaire results concerning all respondents.

Statistical analysis

Resident physicians' knowledge was measured using GM-ITE scores, and the survey was used to measure their perceptions and attitudes. Results of items using the five-point Likert scale were converted into dichotomous variables, with responses ranging from one to three being

interpreted as “not applicable” and from four to five as “applicable.” Categorical variables are presented as proportions.

In the univariate analyses, we performed a chi-square test for categorical variables and either a Mann-Whitney U or a *t*-test for continuous variables, as appropriate. The multivariable analysis model included sex, postgraduate year, university or community hospital, presence of an ID department at medical school, ID department rotation at medical school, AS education at medical school, presence of ID consultation during residency, resident physicians’ exposure to education by ID physicians during residency, AS education during residency, and the presence of AS programs [10,11]. We performed a multilevel analysis assuming that individuals were nested in each hospital (level 1, individual; level 2, hospital).

© 2024 Miwa T et al. *JAMA Network Open.*

eReferences

1. Nishizaki Y, Shimizu T, Shinozaki T, et al. Impact of general medicine rotation training on the in-training examination scores of 11, 244 Japanese resident physicians: a Nationwide multi-center cross-sectional study. *BMC Med Educ.* 2020;20(1):426.
2. Nagasaki K, Nishizaki Y, Shinozaki T, Kobayashi H, Tokuda Y. Association Between Resident Duty Hours and Self-study Time Among Postgraduate Medical Residents in Japan. *JAMA Netw Open.* 2021;4(3):e210782.
3. Nagasaki K, Nishizaki Y, Nojima M, et al. Validation of the General Medicine in-Training Examination Using the Professional and Linguistic Assessments Board Examination Among Postgraduate Residents in Japan. *Int J Gen Med.* 2021;14:6487-6495.
4. Nishizaki Y, Nozawa K, Shinozaki T, et al. Difference in the general medicine in-training examination score between community-based hospitals and university hospitals: a cross-sectional study based on 15,188 Japanese resident physicians. *BMC Med Educ.* 2021;21(1):214.
5. Nagasaki K, Nishizaki Y, Shinozaki T, et al. Impact of the resident duty hours on in-training examination score: A nationwide study in Japan. *Med Teach.* 2022;44(4):433-440.
6. Inoue K, Matsumoto M. Japan's new postgraduate medical training system. *The Clinical Teacher.* 2004;1(1):38-40.
7. Ministry of health, Labour and Welfare. Report on residency in 2019. https://www.mhlw.go.jp/content/10803000/000536692.pdf. Accessed October 4, 2023
8. Ministry of health, Labour and Welfare. Press release on residency in 2023. https://www.mhlw.go.jp/content/10803000/000982320.pdf. Accessed October 4, 2023.
9. Tran K, Hawkins DN, Jacobsen KH. Knowledge, attitudes, and practices related to antimicrobial resistance among undergraduate students at a large public university in 2020. *J Am Coll Health.* 2023;71(6):1873-1878.
10. Nishizaki Y, Shimizu T, Shinozaki T, et al. Impact of general medicine rotation training on the in-training examination scores of 11, 244 Japanese resident physicians: a Nationwide multi-center cross-sectional study. *BMC Med Educ.* 2020;20(1):426.
11. Nishizaki Y, Nozawa K, Shinozaki T, et al. Difference in the general medicine in-training examination score between community-based hospitals and university hospitals: a cross-sectional study based on 15,188 Japanese resident physicians. *BMC Med Educ.* 2021;21(1):214.

© 2024 Miwa T et al. *JAMA Network Open.*
