# Supplementary material for: Infectious Disease Physician Availability and Postgraduate Antimicrobial Stewardship Education in Japan
Source: JAMA Netw Open. 2024 Mar 29;7(3):e244781. doi: 10.1001/jamanetworkopen.2024.4781 (PMC10980957; doi:10.1001/jamanetworkopen.2024.4781)
Supplement: Supplement 2. — Data Sharing Statement [file jamanetwopen-e244781-s002.pdf]

Data Sharing Statement

Miwa. Infectious Disease Physician Availability and Postgraduate Antimicrobial Stewardship Education in Japan. *JAMA Netw Open*. Published March 29, 2024.
doi:10.1001/jamanetworkopen.2024.4781

Data
Data available: No
